# What Leads Indians to Participate in Clinical Trials? A Meta-Analysis of Qualitative Studies

**DOI:** 10.1371/journal.pone.0010730

**Published:** 2010-05-20

**Authors:** Jatin Y. Shah, Amruta Phadtare, Dimple Rajgor, Meenakshi Vaghasia, Shreyasee Pradhan, Hilary Zelko, Ricardo Pietrobon

**Affiliations:** 1 Graduate Medical School, Duke-National University of Singapore, Singapore, Singapore; 2 Research on Research Group, Duke University, Durham, North Carolina, United States of America; 3 Kalpavriksha Healthcare and Research, Thane, India; 4 Department of Surgery, Duke University, Durham, North Carolina, United States of America; University of Oxford, United Kingdom

## Abstract

**Background:**

With the globalization of clinical trials, large developing nations have substantially increased their participation in multi-site studies. This participation has raised ethical concerns, among them the fear that local customs, habits and culture are not respected while asking potential participants to take part in study. This knowledge gap is particularly noticeable among Indian subjects, since despite the large number of participants, little is known regarding what factors affect their willingness to participate in clinical trials.

**Methods:**

We conducted a meta-analysis of all studies evaluating the factors and barriers, from the perspective of potential Indian participants, contributing to their participation in clinical trials. We searched both international as well as Indian-specific bibliographic databases, including Pubmed, Cochrane, Openjgate, MedInd, Scirus and Medknow, also performing hand searches and communicating with authors to obtain additional references. We enrolled studies dealing exclusively with the participation of Indians in clinical trials. Data extraction was conducted by three researchers, with disagreement being resolved by consensus.

**Results:**

Six qualitative studies and one survey were found evaluating the main themes affecting the participation of Indian subjects. Themes included Personal health benefits, Altruism, Trust in physicians, Source of extra income, Detailed knowledge, Methods for motivating participants as factors favoring, while Mistrust on trial organizations, Concerns about efficacy and safety of trials, Psychological reasons, Trial burden, Loss of confidentiality, Dependency issues, Language as the barriers.

**Conclusion:**

We identified factors that facilitated and barriers that have negative implications on trial participation decisions in Indian subjects. Due consideration and weightage should be assigned to these factors while planning future trials in India.

## Introduction

Enrollment and individual subject completion rates are arguably the most demanding [1] phases of a clinical trial. One of the most important factors in a trial's success is potential participants' willingness to enroll. Past studies have identified a wide variety of determinants of this factor including, education [2], [3], age [4], means of communication [5], race [6]–[9], language [10], patient preference for a certain treatment [11], and a multitude of other personal reasons [12]. Although this list might seem comprehensive, these factors cannot be generalized to individuals from different cultures with divergent lifestyles, social environments, religious beliefs, and economic conditions. This is especially problematic because large culturally diverse countries such as India are playing a more significant role in global trials. India is increasingly recognized as a site for health research in part because of it's large population and growing research capabilities [13]. However, to our knowledge no previous studies have consistently addressed the main factors affecting willingness to enroll in clinical trials among Indians, a key determinant in a trial's success.

India's prominence as a suitable location for health research has emerged partly because of its potential for enrolling patients in clinical trials [14], [15]. India has one of the largest enrollment rates in the world, with rates as high as ten times greater than the ones in the United States for selected trials [16], [17]. With a large and heterogenous population, an additional advantage in India is that several diseases have incidence rates similar to other developing and developed countries [18]. However, critics have expressed concerns over the ethical nature of these enrollment rates citing widespread poverty, illiteracy, and lack of understanding regarding their local culture and customs. [19].

Although there is an extensive literature [20]–[22] evaluating the factors promoting and precluding participation in clinical trials among various populations, there are a limited number of studies that focus on understanding the specific attitudes and willingness to participate within the Indian population. Extrapolation of conclusions drawn from other populations to Indian subjects is unreliable, since previous studies have consistently demonstrated important differences across different cultural groups [23]. Examples include cultural differences in trial conditions [24] and financial and social support [25]–[27].

Given the significance of specific cultural factors affecting enrollment and the central role of India in global trials, the objective of our study is to conduct a meta synthesis of qualitative studies that evaluated factors contributing to participation of Indians in clinical trials.

## Methods

### Research question

Our study addressed an important research question as what are the factors, from the perspective of potential Indian participants, that contribute to their participation in clinical trials. To determine factors and barriers contributing to participation in clinical trials, we evaluated qualitative studies and surveys available from the existing literature.

### Ethics

We did not apply for IRB approval as we conducted a qualitative metanalysis based on published literature.

### Search Strategy

Two reviewers (MV, AP) independently performed a systematic search of the following online databases: Pubmed (1985 to 2007), Cochrane (1983 to 2007), Medind (April 1985 to 2007), Scirus (1980 to 2007), Medknow (1986 to 2007), and Openjgate (2000 to 2007). Of these databases, Medind, Medknow, Openjgate are Indian databases.

We initiated our search strategy by using a set of keywords [Appendix S1] relevant to our research question. We used these keywords individually and in different combinations to ensure identification of the relevant literature. Based on the articles found through this initial search we then created a list of related Medical Subject Headings (MeSH) Terms. [Appendix S2] Next we used these MESH terms individually and in various combinations [Appendix S3] using Boolean operators to conduct a detailed search.

To make our search more comprehensive, we also retrieved and reviewed the bibliographic references of all the full texts which were read thoroughly to retrieve the relevant articles. Through this method we identified study titles or abstracts that were related to our topic. We also reviewed articles listed under the “Related articles” link in PubMed. [28]. Finally, we subscribed to RSS (real simple syndication) feeds for the search strategies that we had devised & implemented in online databases, to track new studies (matching our requirements) that might be published after we completed the literature review. Since two reviewers in our team (MV and AP) were fluent in local Indian languages (Hindi and Marathi), we tried to extend the search to databases in these languages. This step was limited by the lack of online publication databases in Hindi and Marathi.

### Selection

We defined selection criteria to filter study articles and shortlist articles that would qualify for the meta synthesis and help us answer our research question. Both reviewers (AP and MV) independently evaluated the study articles that were identified based on our search strategy.

#### Inclusion Criteria

We included studies with the following characteristics: prospective studies; confined to Indians (Indian resident or of Indian origin); using experimental (trials) or qualitative methods (interviews, focus groups, ethnographic, or survey) to collect data; studies whose outcome measures included factors affecting participation of Indians in clinical trials, and full text articles.

#### Exclusion Criteria

We excluded studies with the following criteria: Studies that did not directly evaluate potential participants but rather, evaluated factors influencing participation by analyzing retrospective clinical trial data, studies that evaluated other Asian populations or American Native Indians, unpublished articles, dissertations and abstracts without full text. Our goal was to evaluate the reasons for participation in clinical trials of potential subjects. Since subjects who had already agreed to participate in a trial would have a significant degree of confirmation bias [29], [30], we decided to exclude them from our sample. Articles in languages other than English, Marathi and Hindi were also excluded since the study team was not conversant with other languages.

After the finalization of inclusion and exclusion criteria, one of us (SP) used these criteria to perform an independent search. This reviewer was unaware of the aim of our study and was only given the inclusion and exclusion criteria and was asked to retrieve the related articles. This was an attempt to confirm and cross check the findings of other 2 reviewers (AP and MV) as well as avoid missing any relevant articles.

Finally, we screened the retrieved studies by reviewing them first by title, then by abstract and last by full text, and at each step, excluded those which did not satisfy the selection criteria.

### Hand search

We classified the initial list of articles according to the journal in which they were published, so that we could then identify journals that had published most of the articles in our list. Since the focus of our research question was on Indian potential participants in clinical trials, we decided to concentrate only on Indian journals as we believed that they had a higher chance of publishing articles that matched our requirements. We manually searched through each issue of the following online journals: The Indian journal of medical research (Full text archive, Jan 2003 to Jan 2008) and Indian Journal of medical sciences (Jan 1990 to Jan 2008).

### Communication with authors

In order to confirm that we had identified and retrieved all relevant study articles, we communicated via email with the authors of these study articles to inquire about the existence of any other published studies related to our research question.

### Validity assessment

To assure that all the relevant data were retrieved, we asked a third researcher (SP) to repeat the literature search using our inclusion and exclusion criteria. We neither informed this researcher about the aim of the metaanalysis nor shared the list of studies retrieved by us earlier. Discrepancies were resolved by consensus.

### Data abstraction

Each researcher (MV,AP,SP) independently extracted qualitative data from the included studies into a spreadsheet. All data was segregated under specific headings including: aim, study design, study period, eligibility criteria, geographic location, population characteristics, source of participants, number of participants, number of drop outs, data analysis, outcome measures and qualitative quotes.

### Study characteristics

We collected descriptive data for each study included in our meta synthesis. It included demographic details of participants like age and ethnicity, country where the clinical trial was conducted, intervention details (including study questionnaire) and outcome definitions.

### Qualitative data synthesis

Articles included in our study either reported quotes from their interviews or reported percent of study population that provided a specific response to each survey question. In order to ensure data integrity, we populated the data abstraction sheet with these quotes and percent responses for each question. Each quote and response was then reviewed by three researchers (AP, MV & SP) to generate emerging themes. Disagreement during this process was resolved by consensus. The final abstracted spreadsheet was reviewed by 4th reviewer (RP) to resolve any discrepancies. Next we categorized the themes into 2 groups: Factors favoring participation in clinical trials and factors serving as barriers to participation in clinical trials. We also listed quotes from each study that justified the final themes.

### Percentage retrieval for each of the themes

Although the primary goal of our study was to carry out qualitative data synthesis, we thought it would be equally important and interesting to provide the percentage of participants contributing to each of the theme. We determined the average for similar responses in each study. Next, we calculated the total of similar responses from all studies and reported it as a percentage. This method allowed us to report both quotes from interviews and responses to survey questionnaire, thus minimizing data loss.

## Results

### Search Strategy Results

**Figure 1:**
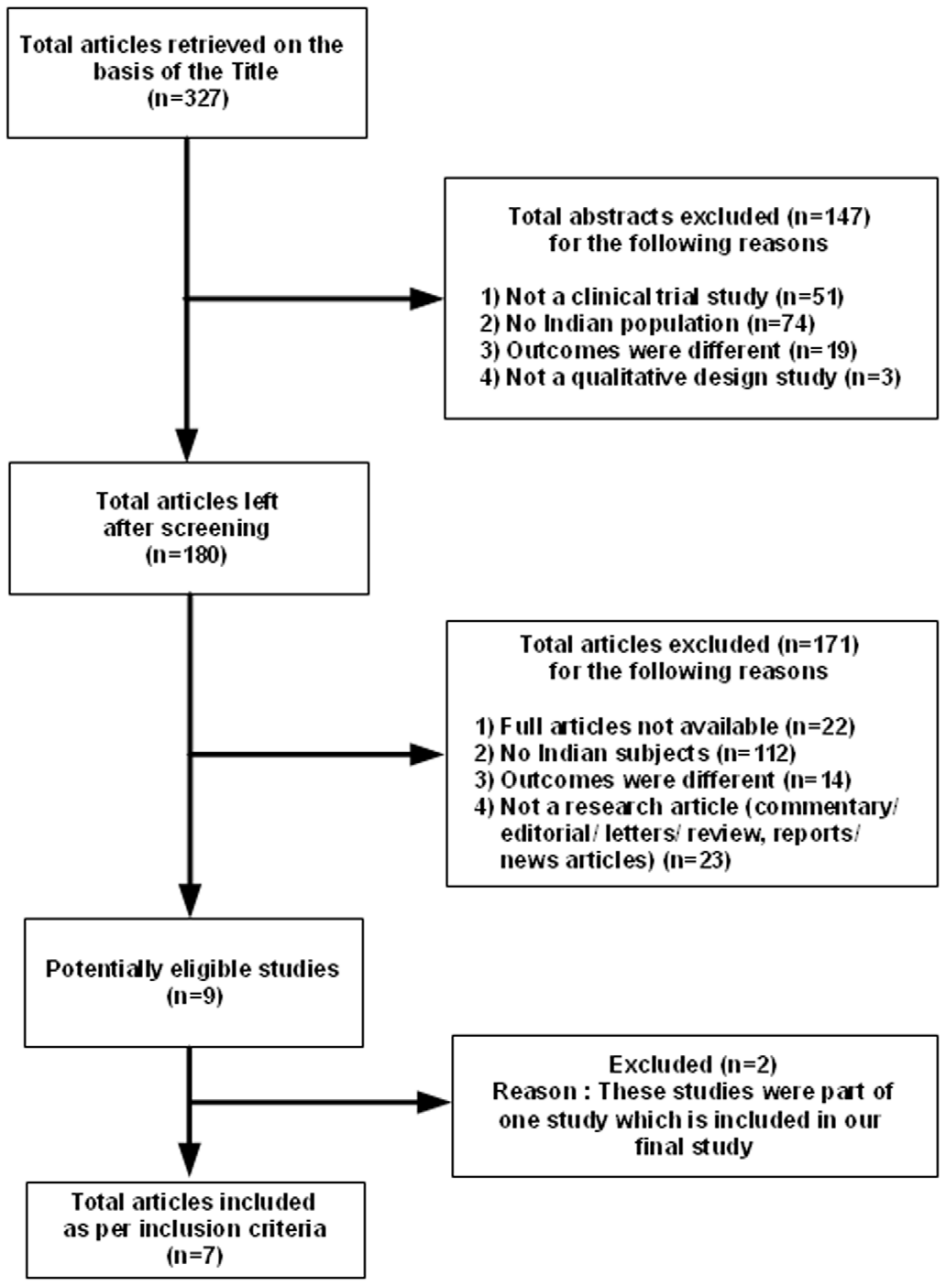
Flowchart with inclusion and exclusion of articles.

The initial review of the literature yielded 327 study articles. After reading the abstracts of these articles, we excluded 147 of them because of the following reasons: 1. They were not clinical trials, 2. They had no Indian population, 3. As the studies did not have desired outcomes, 4. Other study articles were removed because they dealt mainly with educational/prevention programs, screening programs, or medical service camps. We then retrieved and evaluated the full text for the remaining 180 articles, leading to the further exclusion of 171 articles because 1. There full texts were not available, 2. They had no Indian population, 3. As the studies did not have desired outcomes, 4. Other study articles were removed because they dealt mainly with educational/prevention programs, screening programs, or medical service camps. The final list comprised seven studies matching our inclusion and exclusion criteria. This flow chart is summarized in Figure 1.

We retrieved three studies by the same author (Hussain Gambles), two of which were published in year 2004, and the third was published in 2006. Since one of these three studies was a combination of two studies [31], we considered combined study for inclusion in the current meta synthesis and excluded the other two individual studies [32], [33]. Hand search did not yield any studies matching our selection criteria. The final list of seven articles, all using a qualitative research design, is listed under Table S1.

### Study characteristics

Out of the seven studies included in our meta synthesis, four studies were conducted in India and exclusively reported data on Indian population. The remaining three studies were conducted in multiple locations like Singapore, United states (US), United kingdom (UK), France, Poland, Italy, Spain and others. They had a mixed ethnic population. Of the seven studies, three primarily focused on studying subject participation in general while other three focused on HIV vaccine trial participation and one study focused on subject participation in genetic research. The number of Indians enrolled in all the studies is reported in Table S1. The minimum age of participants enrolled in the seven studies was above 15 years except for two studies that did not report this information [24], [34]. We present results derived from the seven studies based on the methods used in each study; namely focus group discussions, semi structured questionnaires and interviews. For example, quotes from focus group discussions and interviews contributed to emerging themes which are presented in table S2 and S3, while data from the studies that used semi structured questionnaire are presented in the form of percentages in the same tables.

### Validity assessment

The list of study articles retrieved independently by the third reviewer matched the ones that the other two reviewers (AP and MV) had retrieved.

### Emerging themes

Thirteen themes were generated after thoroughly reveiwing and analyzing all the seven shortlisted study articles dealing in willingness of Indian subjects to participate in clinical trials. The themes were subdivided into two groups based on factors that facilitated or served as barriers to participation in clinical trials.

### Factors favoring participation (Table S2)

#### Personal Health Benefits 48%

All seven studies contributed to this theme. Potential participants are more likely to participate if they are convinced that the clinical trial output will benefit them in terms of good health, protection from or prevention of some disease. [35] “… *I think a lot of people probably you, would find ‘what's in it for me’. I'm just thinking if my mum and dad were approached, I don't think they would get involved.*” (LI5F3)" [31]. The same is true for relatives who influence decisions on participation. If they are convinced that the participant will personally benefit, they frequently encourage participation. “… *if it makes their wife better and she's going to make the chapattis again, then yes they'll sign it [consent forms] … and again that's from education and that's from their family background, and also what sort of job they do in this country.*” [31]. Free treatment to self or kin, pain relief for self are some other examples that were also valued. Patients with incurable diseases or at the terminal stage of life, choose to participate with the hope that the trials may improve their condition or cure them. [36]. In case of potential participants of a HIV vaccine trial, personal protection from possible HIV infection during and after the trial significantly influenced their decisions. “*Initially it (the HIV vaccine) will give 75% protection. At the end of the research, it will be 100%, hence there is no harm.*” [37]. Notably, married women from the same study cited personal safety from HIV infection as a motivator to participate in trials. “*We may be unaware of the behavior of the men folk. If they had gone astray, there are chances of us also to get infected. By taking the vaccine, this can be prevented*” [37].

#### Altruism 43%

Altruism was present as an emerging theme in all seven studies. Contribution to collective good as well as to the progress of science and medical knowledge also influenced decisions on participation in clinical trials: “*I feel glad to take social risk*”; “*Do something that is good for the world*” [34]. The sense of goodwill by helping in the development of a potential cure or vaccine and thus avert an epidemic was also notable. *“helping to find a vaccine that works”, “helping to stop the epidemic.”* [35]. Finally, helping to prevent a killer disease like HIV and making it preventable were other responses corroborating this theme. “*HIV will become preventable like polio*” [35]. “*HIV/AIDS is a killer disease. It would be wonderful if there is going to be a vaccine for it. We are confident that it would come.*” [37].

#### Methods for motivating participants 34%

This theme, which describes methods to motivate participation in clinical trials, emerged from three out of seven studies. Respondents communicated their preference for trials initiated by research institutes or government agencies. They were more inclined to participate, if information on clinical trials was provided through government owned television channels. The same was true in case of HIV vaccine trials where it was apparent that potential participants placed a lot of faith in government endorsement and were more likely to believe that it was safe to participate. “*If it is done through the government, many people would come forward to take it*”. *"The government is never wrong. Therefore, if the government endorses a vaccine, it will surely be safe. So we can take the vaccine without fear.”* [37].

Dissemination of clinical trial-related information during healthcare camps could also influence potential participants: “*Sometimes they organize specialists to come in so we can talk about our health problems. If they talked about clinical trials and medical research I think many people would take an interest and be willing to participate*.” (LI20M3)" [translated from Gujarati] [31]. Other motivating factors ranged from advice by regular/usual physician (51/128, e-mail notifications  = (47/128, traditional media (eg. Newspapers, magazines, TV, radio) (42/128), a relevant advocacy organization/patient support group (26/128, internet websites (53/128, Harris interactive [38] (60/128) and family/friends (28/128). Widespread and educative advertisements especially in places of high public thoroughfare were also recommended by potential participants of a HIV vaccine trial. “*The advertisement for the polio vaccination is widespread and educative. HIV vaccination may be done on similar lines. It would be beneficial if HIV vaccine is also given in railway stations and bus stops.*” [37].

#### Source of extra Income, 31%

Responses that pointed toward this theme were noted in four of seven studies. Willingness to participate was more pronounced if monetary remuneration was involved. Respondents expected an incentive for clinical trial participation in terms of monetary gains or incentives such as gifts and insurance coverage. Additionally potential participants in HIV vaccine trials demanded that the monetary/insurance benefit be guaranteed to them or next of kin in case of death. “*We want a written guarantee plus insurance policy. The document should specify that, in the event of death (of the person after taking the vaccine), his family would be given full support.” "Security assurance that in the case of amiss, families would be taken care of.”* [37].

#### Detailed knowledge 21%

The need to keep participants informed about the potential risks involved in clinical trial participation was reinforced in multiple studies. Respondents indicated that knowledge about the involvement/non involvement of risk and information on current medication tended to influence their decisions. This was apparent when participants asked for more information before they agreed to, for example, donate blood specimens. In case of HIV vaccine trials, participants stressed on the need of education, information and clarification about the nature and characteristics of HIV vaccine. “*The ELIZA test will be positive due to vaccination. We should tell those persons who had taken the vaccines that the positivity would disappear after some time”.* [37]. Specifically they wanted answers to questions like the frequency, site of administration, period of protection and effectiveness of HIV vaccines. “*Community members would want to know about the frequency of the vaccines and the site administered, how it might impact marriage, how long the HIV positive result will last, how long will the vaccine protect for, and will the vaccine be effective.”* [37]. Finally, terminology, language and style played an important role in communicating study-related information to the potential clinical trial participants: *“I understood bits of it, some things I didn't understand. The second time I went I took my daughter with me. She explained what he said and that they will offer to get somebody to translate for me. When I visit the doctor I occasionally take my daughter because of the terminology used.”* [31].

#### Trust in Physicians 8%

This theme was present in five out of seven studies. Respondents in the qualitative studies expressed the important role of family physicians/general practitioners (GP) in the clinical trial participation - decision-making process. In comparison to the educated, the uneducated are more inclined to follow their GP's advice in relation to clinical trial participation. This can be seen from the quote *“… well I think especially the ones that are uneducated, they're easily persuaded, easily persuaded because I mean, okay, I regard my GP pretty highly, okay, but I also realise that GP just offer an opinion. Whereas if I was uneducated, GP's God … the Asian community always looks at, well doctors being the top profession, the top everything. Whatever they said they will believe they will do it, you know. I mean I would possibly be inclined, but they would definitely do it, I reckon.*” [31].

### Factors serving as barriers to clinical trial participation (Table S3)

#### Mistrust of trial organizations, 26%

This theme emerged from responses observed in four out of seven studies. Respondents made personal inferences about the main aim of some trials and felt they were “treated as guinea pigs.” Also, they felt there was always a possibility of placebo administration that would not benefit them in any way, indicating that mistrust was a main factor influencing their refusal to participate. On the other hand, it was observed that family doctors enjoyed their patients' trust. This was evident from the potential participants' desire to consult their family doctors to advise them on safety in relation to participation in trials.

#### Concerns about efficacy and safety of trials, 21%

This theme was present in three out of the seven studies. Potential participants voiced their concerns about safety procedures as well as possible side effects and health risks that might be associated with clinical trials. They were also concerned about the unproven nature of the therapy that they would be subjected to. Specifically in relation to vaccine trials, patients reported concerns related to unknown efficacy and long-term adverse effects.

#### Dependency Issues, 19%

Responses from two out of seven studies gave rise to this theme. Respondents frequently depended on family, friends or society in general to guide their decision to participate in clinical trials. Participation of a friend served as an example and encouraged participation because it made them feel more comfortable and as if they were not alone in their decision: ‘*Can come if a friend comes’ and* ‘*Cannot come alone*.’ Finally, the need to consult children, spouse or other family members was strongly felt before arriving at a decision. *‘Have children at home…*’; ‘*Do not have knowledge, will ask husband.…’; ‘Has to see what husband says…’; ‘Husband does not allow, then I will not come…’* [34]

#### Loss of Confidentiality, 17%

This theme emerged from responses noted in four out of seven studies. Respondents were concerned about privacy safeguards and cited the potential negative impact of loss of confidentiality on personal life, marriage, insurance and employment when describing reasons for not participating in clinical trials. Respondents also feared that personal health matters would be disclosed in a clinical trial which may end up causing personal harm to them. Accordingly they placed a lot of importance on keeping their personal information confidential. “*If you are checking whether I have the gene for cancer or heart diseases, it is ok. However, my privacy is very important especially if you are checking for a gene for a personality disorder. I dont want people poking their noses into my family“ - Indian female manager*.” [39].”

The same concern was echoed by potential participants of HIV vaccine trials. *Notably the social stigma attached to HIV/AIDS made them highly concerned about the confidentiality of their personal information. “Friends will suspect us if we undergo HIV testing,” and* “*People do not consider HIV/AIDS as an ordinary/another disease. The stigma attached to the disease is persisting still. Other people may get to know about the result when the trials are being conducted. No one will come forward to take part in the vaccine trial if there are going to be chances that others/outsiders may know the result. Why? Even I shall not come. Men having sex with men are not given/accorded status in society. We agree that you are working for a worthy cause. But if everyone (public/outsiders) is going to know about their HIV status, none of the men having sex with men will agree for the trial. It would be better if the stigma attached to HIV/AIDS were removed from the minds of the public before the vaccine is introduced.*” [37].

#### Trial burden, 11%

Five out of seven studies contributed to this theme, which primarily related to the challenges and difficulties involved in clinical trial participation that ultimately burden the subject. These include trial procedures and protocol that have the potential to disrupt routine life and cause inconvenience to a subject. For example, respondents cite time constraints, travel, intake of additional and unnecessary drugs in four studies. On the other hand, trials that were deemed to be more convenient and less disruptive of routine life enhanced participation [36]. While commenting on retention in HIV vaccine trials, potential participants showed a preference for additional facilities that would reduce the impact of trial participation on their daily lives. Examples such as a paid leave from office, location of trial venue near the house and short duration of trials were noted. [37]. Finally, frequent blood specimen collection was perceived to be inconvenient and in conflict with busy schedules: ‘*How often will blood specimens be taken? If you come very often, it will cause us a lot of inconvenience. We are all very busy.*’ (Indian Female factory worker) [39].

#### Psychological reasons, 6%

Numerous sub themes dealing with psychological factors influencing subject decisions to participate in clinical trials contributed to the formation of this theme. These factors ranged from plain fear to fear of injection/stigma/blood tests as well as disinterest in participation: *“Well I mean it's just, you know, sort of fright, that's all I can think of really, fright. Fright and the dangers I can associate with it, that's all. I mean, okay, it's selfish in a way because other people try it, trial themselves for me.”* [31]. Some participants were anxious about the possibility of detection of something new and unpleasant that might result in an incurable disease while others were plainly not interested.

#### Language 1%

This theme was found in only one out of seven studies. Respondents preferred the communication of clinical trial information in a simple and lucid manner. Additionally, language barriers frequently caused respondents to have difficulty understanding the procedures, safety, and benefits of the ongoing clinical trials, which subsequently resulted in neglect and non-participation in clinical trials: “… *it's hard to understand the language because it's complicated in the words that they [doctors] use. And we don't know any different or how to go about arguing with him.”* [31] and “*did not understand”* [9].

## Discussion

Our meta analysis retrieved, selected and reviewed qualitative studies that evaluated the factors influencing participation of Indian subjects in clinical trials. The meta synthesis of factors cited by Indian subjects provides a better picture of their mindset by revealing what favors or hampers their decision to participate in clinical trials. The emerging themes from our study broadly fall under two categories: Factors favoring participation and factors restricting participation. The former includes the themes: personal health benefits, altruism, trust of physicians, source of extra income, detailed knowledge about trials and methods for motivating participants. On the other hand the latter category includes: mistrust on trial organizations, concerns about efficacy and safety of trials, psychological reasons, trial burden, loss of confidentiality, dependency issues and language. Our study also tried to derive the quantitative data in terms of percentage of population for each of the themes. Though these figures may not be precise and accurate they do represent the broad percentages and would certainly needs attention as to which of the theme might need more focus in terms of any rectification that might have to be implemented either to eliminate barriers or to encourage participation.

Our study focused specifically on Indian population for the following reasons: 1. India as a part of the BRIC nations is the current hotspot for the conduct of clinical trials. 2. Its huge and diverse (racially and ethnically) population is considered favorable for clinical trial implementation because it ensures rapid enrollment and enhances the generalizability of results. Additionally racial and ethnic groups have their own unique ways of looking at, interpreting and deciding on clinical trial participation on account of numerous factors such as their beliefs, religion, knowledge about experimental studies [40]. Therefore, it is essential to understand and evaluate factors that influence their decisions to participate in clinical trials.

Indians were highly willing (prevalence = 47%) to participate in clinical trials when they were convinced that there were personal healthcare advantages. The influence of personal benefit on important decisions like participation in a clinical trial is a natural human tendency and has been confirmed in all major ethnic groups [40]. It can take a variety of forms such as access to free medicines and latest treatments [41]–[44], possible chance of cure by the trial intervention [20], possible relief by trial intervention [45] and possible protective benefit through vaccine trials are some of the most common forms. On the other hand, personal benefit does not always spur participation in trials as apparent from factors like 'non availability of alternative treatments' cited in previous studies [25], [31]. It is important to note that although personal health benefit is often thought of as an individual driver of participation, it may often be affected by cultural, socio-economic and healthcare conditions prevailing in a country. For example, populace hailing from low socio economic background and with little access to quality healthcare are attracted by the free treatment and financial incentives provided in clinical trials. In contrast to the factor 'personal health benefit,' 43.36% subjects also believed in altruism which is a selfless wish to benefit peers, society and science. For example, a patient may participate not only because they hope to be cured but also because they want others to be cured. Previous studies have also simultaneously encountered these contrasting factors. [41], [42]. Altruistic motivations for participating in trials are evident when healthy volunteers participate in clinical trials with the aim of benefiting ailing patients around the world. This inclination has been noted in a number of ethnic groups [31], [41], [44], [46]–[48] and among HIV patients who are usually influenced by safety and personal benefit concerns [42], [49]–[51]. Apart from these two factors, the theme – ‘trust in physician’ (7.9%) reflects the fact that patients frequently rely on their doctors advice while deciding to participate in clinical trials. Patients consider their physicians as their health guide and frequently trust them blindly either out of respect, feeling of indebtedness or based on the belief that their physician would never misguide them. As a result, when physicians ask their patients to participate in a clinical study, they are unlikely to refuse. Along similar lines, previous studies mention the role of 1. physician recommendation, communication and encouragement and 2. sharing trial information with family physicians [20], [25], [48], [52]–[55]. Previous studies also confirm that a converse relationship (mistrust on physician) has a significant impact on trial participation [20], [56].

Though personal health benefits and altruism were the major influencing factors, monetary gain also emerged as a significant theme in our study. It has also been documented in other similar studies [57]. In a developing country like India where poverty is rampant, participating in trials that offer monetary incentives is an extra source of income. Even when the trial does not offer any monetary compensation, the free care and treatment serves as a strong attraction for patients who otherwise can not afford the cost of treatment. Given the influence of an incentive, its ability to distort potential participants' judgment towards trial participation is significant [58]. As per the metasynthesis, “detailed knowledge” about the objectives and procedures in the clinical trial enhances trial participation. This is empirically achieved through an informed consent sheet and a verbal explanation to rule out possible misconceptions about the trial. Lack of detailed information can raise concerns [20], and lead to non participation, while hiding any information or furnishing incomplete information would amount to violation of the ethical conduct of the trial. Motivational methods like newspaper, tv ads [43], newsletters, mail shots [1] and audio visual media can further enhance recruitment by increasing awareness, clarifying misconceptions if any as well as educating participants about clinical trials. Although it was predominant across all studies, one study made a contrasting observation: complete or partial disclosure of the consent process (where patients are provided detailed trial information) did not alter the number of subjects consenting to participate [24]. Rather subjects relied on the explanation and answers furnished by the researchers. Factors like treating physician, poverty and illiteracy might have led to this observation. Summing up, factors like personal health benefit, altruism and monetary gains being subjective lie in the personal decision making domain. On the contrary themes like trust in physicians and detailed knowledge suggest that physicians play an important role in recruitment and they should be well trained and knowledgeable about the trial.

While trust in physician favored trial participation for 7.9% of the subjects, mistrust on physicians and researchers was also expressed as a barrier by 26.27% of the subjects. Other factors like lack of faith and fear towards the healthcare system [23], [59], [60], the notion of 'guinea pig treatment' [40] and mistrust in clinical research [23], [48], [60]–[63] have been shown to negatively influence subject participation in clinical trials. It is important to note there can be a different effect on trial participation depending on whether the treating physician, trial recruiters or researchers were involved in recruitment. While trial recruiters may not be trusted as much as physicians, they often have more training and time for recruiting potential trial participants. Differences in the perceptions about trust or mistrust on trial organization, researchers or pharmaceutical industries can be due to multiple ethnic variations, previous experience in trial participation, language and literacy [64]–[65]. Promoting trust and identifying other sources of mistrust in the trial organization are possible solutions that can enhance subject trust [66], [67]. A lack of detailed knowledge, motivation methods and proper communication methods can significantly enhance the mistrust of subjects in trials. It can also contribute to the theme – ‘concerns about safety and efficacy of trials’ where unproven efficacy, side effects and health risks of experimental treatment may deter subjects from participating in clinical trials. Similar findings have been documented in the Chinese population [25], in cancer trials [52] and placebo controlled trials [44]. The metasynthesis also identified 'psychological reasons' that deter subjects from trial participation. Some examples were 1. preconceived notions about various aspects of clinical trial participation such as pain and number of tests that they (subjects) would have to undergo and 2. the social stigma involved. This is more pronounced in the case of HIV vaccine trial participation where participants may 1. fear serious adverse health consequences, 2. fear the possibility of getting infected due to vaccination and 3. fear on being denied health insurance coverage [68]. Apart from the misconceptions and preconceived notions, some subjects also found trial participation to be a burden as it requires extra time and effort. Participation often meant that subjects would be required to make additional visits to the doctor, spend more time traveling and consume additional drugs. When this burden is decreased, Indian subjects are more likely to participate in clinical trials. Other studies have noted that living at a distance from the trial site and having to participate in a trial with multiple follow up visits tend to be burdensome for the subject and acts as a barrier to clinical trial participation. [21].

In addition to the factors listed above, 17.08% of Indian subjects expressed 'privacy and the negative impact of loss of confidentiality' as an important concern. Maintainence of confidentiality in order to ensure integrity and privacy of participant information has been emphasized and acknowledged in previous studies [69]. Additionally, confidentiality issues and breach of confidentiality have been a concern in genetic association studies [26] as well as in cancer clinical trials [21]. On one hand when subjects were concerned about the maintenance of privacy and confidentiality, a sizable number (18.65%) seeked advice and were dependant on relatives, peers and friends while making their decision. In India and other parts of Asia, patients frequently discuss health issues with family and friends which contradicts the American culture, where doctors typically do not even discuss or disclose a patient's health information to their family members [25]. The involvement of family members in decisions about healthcare is a cultural factor that has also been identified in studies of Chinese subjects [25]. In contrast, studies of African Americans have shown that healthcare decisions are highly individualized and autonomous and the involvement of family members can actually inhibit, rather than promote participation in clinical trials [8], [70]–[72]. Conveying the right information in a simple and clear manner as well as in a language that they understand is important for ensuring optimal delivery of clinical trial information to subjects. Indian subjects in our meta synthesis confirmed this fact when they expressed language and miscommunication as potential barriers to trial participation. Language has also been identified as a barrier by Chinese subjects [25], older south asian population in UK [31] as well by other populations [73], [74]. In fact, it has been argued that language barrier can lead to mistrust which can negatively influence trial participation. In order to avoid language barriers, several methods have been introduced such as: translation into the local language and the use of translators to converse with potential participants in their local languages. For example, since the primary language of the consent form is usually English, several translations of the consent form are made as per the local requirements. This does facilitate understanding of the study by literate people, although it remains unclear how much they actually understand. It has also been emphasized that consent forms should be written for lay man and avoid highly technical language. However, in practice this is not always possible and the use of specialized terminologies in the consent form may cause confusion for the subjects.

Because results from qualitative studies are conducted with an aim to reach depth rather than external validity, the combination of results needs to be subsequently verified through studies evaluating external validity such as population-based surveys. Also, since India is a very diverse country with a variety of ethnicities, religious beliefs, and cultural heritages, these results might vary across different groups, more granular investigations being required once more studies are made available. One other limitation of our study is non availability of full texts of twenty two articles which we had to omit without any option with us.

Our meta analysis identified factors facilitating trial participation specifically for Indian subjects. In order to maintain its status as a hub for clinical trials, India needs to take steps to ensure adequate trial participation. Insights into the mindsets of Indian subjects in relation to trial participation can guide investigators and sponsors when they plan trials in the future. Non consideration of these factors during the planning stage may lead to delays in the trial enrolment and subsequent implications on trial completion and costs. There are several other reasons and factors associated with the decision to participate in a trial that have been expressed by other subjects belonging to different ethnic groups from various parts of the world. However, they remain specific to the region, ethnicity, the disease under study like HIV, cancer, type of intervention-invasive/non invasive, type of study design-use of placebo or any other.

## Supporting Information

Table S1 Characteristics of studies included in metaanalysis.
(0.04 MB DOC)

Table S2 Factors favoring participation in clinical trials.
(0.04 MB DOC)

Table S3 Factors serving as barrier to participation in clinical trials.
(0.04 MB DOC)

Appendix S1 List of keywords for database search.
(0.03 MB DOC)

Appendix S2 List of mesh terms collected from different articles and used to search databases.
(0.03 MB DOC)

Appendix S3 Mesh terms and subheading combinations used for database search.
(0.03 MB DOC)
