# Supplementary material for: What Leads Indians to Participate in Clinical Trials? A Meta-Analysis of Qualitative Studies
Source: PLoS One. 2010 May 20;5(5):e10730. doi: 10.1371/journal.pone.0010730 (PMC2873955; doi:10.1371/journal.pone.0010730)
Supplement: Table S1 — Characteristics of studies included in metaanalysis. (0.04 MB DOC) [file pone.0010730.s001.doc]

**Table S1.** **Characteristics of studies included in metaanalysis**

| **Study title** | **Country** | **Total Subject Population (Indians)** | **Intervention** | **Age group** | **Factors evaluated** |
| --- | --- | --- | --- | --- | --- |
| A pilot study on willingness to participate in future preventive HIV vaccine trials | India | 112 (All Indians) | Structured Questionnaire method | Above 15 yrs | - Willingness of populations at risk to participate in future preventive HIV vaccine trials (HIVVTs) - Factors that enhance or deter from participation |
| Participation in Clinical Trials Lower in Europe and India than in the United States | US | 2935 (128 Indians) | Online interview | 18 yrs and above | - Opportunity and Participation in Clinical Trials - Reasons for participation and likelihood of future participation - Factors Very Likely to Influence Participation - Risks and Benefits of Participating in Clinical Trials - Informed Consent Process - Risks and Benefits of Participating in Clinical Trials - Pros and Cons of Clinical Trials - How People Learn and How They Would Prefer to Learn About Clinical Trials |
| Concerns over participation in genetic research among Malay-Muslims, Chinese and Indians in Singapore: a focus group study | Singapore | 98 (31 Indians) | Focus Group Discussions | 18 yrs and above | - Concerns over participation in genetic research |
| Correlates of HIV vaccine trial participation: an Indian perspective | India | 349 (All Indians) | Semi-structured questionnaire | Not mentioned | - Concerns and the level of preparedness for HIV vaccine trials among the persons with low- and high risk for HIV infection |
| Involving South Asian patients in clinical trials | UK(Leeds and Bradford areas of England) | 100(20 Indians) | Semi-structured interviews | ≥18 years old | - South Asian people perception regarding trial involvement - Risks and benefits involved |
| Recruitment of subjects for clinical trials after informed consent: does gender and educational status make a difference? | Pondicherry, South India | 152 (All Indians) | Questionnaire | Information not present in publication | - Approval of patients to participate in a trial after receiving either complete or partial information regarding a trial. - Gender or educational status-related differences in opinion of participation - Reasons for consenting or refusing - Depth of understanding of informed consent |
| Perceptions of a Community Sample about Participation in Future HIV Vaccine Trials in South India | India | 112 (All Indians) | Focus Group Discussions | 18–55 years of age | - Willingness of high-risk groups to participate in a HIV vaccine trial - Factors that might impact these individuals’ participation in a vaccine trial - Potential impact of HIV vaccine trial participation on risky drug and sexual behavior |
