# Supplementary material for: What Leads Indians to Participate in Clinical Trials? A Meta-Analysis of Qualitative Studies
Source: PLoS One. 2010 May 20;5(5):e10730. doi: 10.1371/journal.pone.0010730 (PMC2873955; doi:10.1371/journal.pone.0010730)
Supplement: Table S2 — Factors favoring participation in clinical trials. (0.04 MB DOC) [file pone.0010730.s002.doc]

**Table S2. Factors favoring participation in clinical trials**

| **Personal health Benefits** | **Altruism** | **Methods for motivating participation** | **Source of extra income** | **Detailed knowledge about trials** | **Trust in Physicians** |
| --- | --- | --- | --- | --- | --- |
| Less chance of getting infected with HIV | Level of Protection | Rely on Research institutes for providing information regarding safety | Money/Gift | If they know the risks associated with the treatment | Preferred to rely on specific health care providers (family doctors, counselors) for providing information regarding safety |
| Protection from HIV | Somewhat Likely belief in the success of AIDS vaccine | The government agencies or the government television news channel | Insurance | If they knew that no risks are involved | Doctors only do good |
| HIV vaccine is very important for self | Vaccine for control of AIDS epidemic in India | Regular/usual physician | If money is received for participation | Provide information on current medications |  |
| HIV vaccine is somewhat important for self | Altruism | Email notifications | Money is provided | Knowledge about HIV/AIDS and Vaccines |  |
| If they had a terminal illness | If the drug/treatment would help someone else in the future | Traditional media (eg. Newspapers, magazines, TV, radio) | Free medication is provided |  |  |
| If they thought the drug would cure them | Help advance science and find a cure for diseases / conditions | Internet websites |  |  |  |
| If an active drug is received instead of placebo | May help to save lives | Mail |  |  |  |
| If the drug/treatment would help me | Allows to help others with the condition | Harris interactive |  |  |  |
| If there were no other medical options  available to me | Allow medical team to find an effective treatment | Family/Friends |  |  |  |
| May help themselves with the condition | Help medical community | Awareness of the HIV Vaccine Trials Preparations in India |  |  |  |
| Relief of pain | Willingness is important for the common good of India | Awareness of Vaccination Priorities |  |  |  |
| Level of Protection | Help researchers prevent HIV/AIDS | Facilitators of Participation |  |  |  |
|  | There will be an effective HIV vaccine in a few years |  |  |  |  |
|  | HIV will become preventable like polio |  |  |  |  |
|  | Even if the vaccine does not work, help researchers find an effective vaccine |  |  |  |  |
|  | Help researchers prevent HIV/AIDS |  |  |  |  |
| 48% | 43% | 34% | 31% | 21% | 8% |
