# Supplementary material for: What Leads Indians to Participate in Clinical Trials? A Meta-Analysis of Qualitative Studies
Source: PLoS One. 2010 May 20;5(5):e10730. doi: 10.1371/journal.pone.0010730 (PMC2873955; doi:10.1371/journal.pone.0010730)
Supplement: Table S3 — Factors serving as barrier to participation in clinical trials. (0.04 MB DOC) [file pone.0010730.s003.doc]

**Table S3. Factors serving as barrier to participation in clinical trials**

| **Mistrust on trial organizations** | **Concerns about efficacy and safety of trials** | **Dependency Issues** | **Loss of Confidentiality** | **Trial burden** | **Psychological reasons** | **Language** |
| --- | --- | --- | --- | --- | --- | --- |
| Worry about mode of treatment i.e. whether given vaccine or placebo | Side effects of vaccine/not sure of safety Long-term effect of vaccine | Difficult to decide in anticipation | Effect on insurance, marriage or getting a job | Time constraint | Fear of injection | Do not understand |
| Do not want to take drugs without treating doctors permission | Concern about safety procedure | Lack of privacy at home | Privacy concerns | Effect on travel | Fear of stigma |  |
| Are like guinea pigs | Unknown efficacy of vaccine | Lack of supportive network/family commitments |  | Did not want to take new drugs | Afraid of tests |  |
| Are taking a gamble with their health | Concern about effects of a HIV vaccine on participant's lives | Peer family pressure |  | Unnecessary  tablets | Not interested |  |
|  | Possible unknown long-term side effects of vaccine | Social support |  | Wanted to go home |  |  |
|  | Possible side effects | Not able to make independent decision |  |  |  |  |
|  | Health risks |  |  |  |  |  |
|  | Unproven therapy |  |  |  |  |  |
| 26% | 21% | 19% | 17% | 11% | 6% | 1% |
