# Supplementary material for: What Leads Indians to Participate in Clinical Trials? A Meta-Analysis of Qualitative Studies
Source: PLoS One. 2010 May 20;5(5):e10730. doi: 10.1371/journal.pone.0010730 (PMC2873955; doi:10.1371/journal.pone.0010730)
Supplement: Appendix S1 — List of keywords for database search. (0.03 MB DOC) [file pone.0010730.s004.doc]

***Appendix S1***

**List of keywords** **for database search**

Subject participation in clinical trials, patient participation, research participation, South Asian/Indian participation,willingness to participate in clinical trials, clinical trials and India/South Asian, perceptions and clinical trial, attitudes and clinical trials/participation.
