# Supplementary material for: What Leads Indians to Participate in Clinical Trials? A Meta-Analysis of Qualitative Studies
Source: PLoS One. 2010 May 20;5(5):e10730. doi: 10.1371/journal.pone.0010730 (PMC2873955; doi:10.1371/journal.pone.0010730)
Supplement: Appendix S2 — List of mesh terms collected from different articles and used to search databases. (0.03 MB DOC) [file pone.0010730.s005.doc]

## Appendix S2

**List of mesh terms collected from different articles and used to search databases:**

Patient participation, research subjects, subject participation, research participation, willingness to participate in clinical trials, South Asian/Indian participation in trials

 Clinical Trials as Topic/psychology*· Communication Cultural Diversity· Decision Making Barriers· Cultural Diversity· Decision Making· Health Knowledge, Attitudes, Practice*· Humans

· India/ethnology· Interviews as Topic· Socioeconomic Factors· Urban Population· Health Knowledge, Attitudes, Practice*· Minority Groups/education· Minority Groups/psychology*· Motivation· Patient Acceptance of Health Care/ethnology*· Patient Selection*· Qualitative Research· Research Design/standards· Trust· Research Subjects/psychology*· Patient Participation/psychology*· Asia/ethnology· Asian Continental Ancestry Group/psychology*· Attitude to Health/ethnology*
