# Supplementary material for: What Leads Indians to Participate in Clinical Trials? A Meta-Analysis of Qualitative Studies
Source: PLoS One. 2010 May 20;5(5):e10730. doi: 10.1371/journal.pone.0010730 (PMC2873955; doi:10.1371/journal.pone.0010730)
Supplement: Appendix S3 — Mesh terms and subheading combinations used for database search. (0.03 MB DOC) [file pone.0010730.s006.doc]

## Appendix S3

**Mesh terms and subheading combinations used for database search:**

"Asian Continental Ancestry Group/psychology"[Major] AND "Patient Participation"[Mesh], "Patient Participation"[Mesh] AND "Asian Continental Ancestry Group/psychology"[Mesh],

"Patient Participation"[Mesh] AND "India"[Mesh], "Research Subjects/psychology"[Major] AND "Clinical Trials as Topic"[Mesh], "Research Subjects/psychology"[Mesh] AND "Patient Participation"[Mesh], "Qualitative Research"[Mesh] AND "Patient Acceptance of Health Care/ethnology"[Mesh:NoExp], ("Decision Making"[Mesh] AND "Patient Participation/psychology"[Mesh]) AND "Clinical Trials as Topic"[Mesh], ("Qualitative Research"[Mesh] AND "India/ethnology"[Mesh]), "India/ethnology"[Mesh]) AND "Patient Participation"[Mesh]

 "Patient Participation/psychology"[Mesh] AND "Qualitative Research"[Mesh], ("Patient Participation/psychology"[Mesh] AND "Socioeconomic Factors"[Mesh], ("Motivation"[Mesh] AND "Patient Participation/psychology"[Mesh]) AND "Clinical Trials as Topic"[Mesh] ("Motivation"[Mesh] AND "Patient Participation/psychology"[Mesh]) AND "Qualitative Research"[Mesh]

"Qualitative Research"[Mesh] AND "Asian Continental Ancestry Group/psychology"[Mesh] ,("Qualitative Research"[Mesh] AND "Patient Acceptance of Health Care/ethnology"[Mesh]

("Qualitative Research"[Mesh] AND "Patient Participation"[Mesh]) AND "Minority Groups/psychology"[Mesh] ,"Patient Participation"[Mesh] AND "Minority Groups/psychology"[Mesh]

"Patient Participation/psychology"[Mesh] AND "Asian Continental Ancestry Group/psychology"[Mesh] ,("Patient Participation"[Mesh] AND "Asian Continental Ancestry Group/psychology"[Mesh]) AND "Attitude to Health/ethnology"[Mesh], ("Patient Participation"[Mesh] AND "Health Knowledge, Attitudes, Practice"[Mesh]) AND "Qualitative Research"[Mesh]

("Patient Participation"[Mesh] AND "Health Knowledge, Attitudes, Practice"[Mesh]) AND "Clinical Trials as Topic"[Mesh], ("Patient Participation"[Mesh] AND "Clinical Trials as Topic"[Mesh]) AND "India/ethnology"[Mesh]
